# Notes and News

**Published:** 1891-04-25

**Authors:** 


### Notes and News.

Collected and Communicated.

H.R.H. the Duke of Connaught, will preside at the dinner in aid of the funds of the North-Eastern Hospital for Children, to take place on May 15th, at the Holborn Restaurant. Nearly £4,000 is required to pay off the debt at present owing, and to meet the outlay incurred in improving the sanitary arrangements of the hospital.

Special courses of lectures have been opened in the Alexandra Hospital, in St. Petersburg, for ladies who are working there as chemists' apprentices. The lectures are to be delivered by professors of the military medical academy, and will include instruction in pharmacology, botany, and chemistry. The necessary funds have been provided by two anonymous members of the Town Hospital Commission.

The systematic culture of the voice has hitherto escaped attention in technical education, but the new prospectus of the Forsyth Technical College announces that Professor d'Odiardi will give practical lessons and lectures on the hygiene-management of the voice in speech and song. His system, which is unique and based on scientific study of the vocal organs, will include the art of improving the health, by the mode of breathing, speaking, and singing. This latest addition to the course of technical instruction should prove both popular and valuable.

The foundation stone of the new wing, which is being constructed for the National Orthopædic Hospital, was laid by the Marquis of Lorne, on the 14th inst., in the presence of a large number of friends. The hospital was founded in 1838, and has done a large amount of good, but the need for increased accommodation, especially in the section devoted to children, is very great. The cost of the new wing is estimated at £7,000 towards which sum £4,500 has already been subscribed. The committee earnestly hope to obtain the balance before the completion of the building.

Mr. B. L. Cohen presided at the festival dinner of the North London Consumption Hospital, at the Hotel Métropole, on Wednesday (15th inst.), and stated the case of that institution very effectively. The hospital is not local or metropolitan but national, and patients from all parts of the kingdom were admitted. Last year they received 396 in-patients and 3,218 out-patients. The number of deaths from consumption in London alone was very large, and the total existing accommodation for treating this terrible disease was altogether inadequate, and the Committee desired to raise £6,500 for the purpose of enlarging the hospital, which, when complete, would accommodate 120 patients. Towards this £2,000 had already been subscribed, and it was hoped the whole amount would be raised before the company separated. At the close of the meeting subscriptions to the amount of £2,427 were announced by Mr. Hill, the indefatigable Secretary of the institution.

The sixth meeting of the eighth session of the Hospitals Association will be held at Guy's Hospital on Wednesday 29th inst., when Mr. Lushington, Treasurer of Guy's Hospital, will be in the chair. Papers will be read by Dr. Steele on "Hospital Sanitation, with an account of the Victoria Infirmary, Glasgow"; and by Mr. Saxon Snell on "An Enquiry into the Structural Arrangements of the Nursing Departments of Hospitals." Tickets may be obtained on application to the Secretary of the Association, 140, Strand, W.C.

The sixteenth annual report of the St. John's Home for Convalescent and Crippled Children, at Brighton, records that the last year has been a somewhat eventful one, and a good deal has been done, which it is hoped will be for the benefit of the inmates; 139 children have been admitted during the last nine months, and with the exception of a slight epidemic of measles, their health has been good. The receipts, which include £60 from a concert, and £102 1s. 6d., the proceeds of a ball, exceeded the expenditure by £254 16s. 6d.

The annual meeting of the Society for the Study of Inebriety was held on April 7th, when Dr. Norman Kerr gave an interesting address on medical jurisprudence, pointing out the necessity for better and more certain methods of dealing with the criminal drunkard. There is urgent need for a reformed jurisprudence according to Dr. Norman Kerr, and he aptly cited some recent trials with inebriate complications in support of his contention, the audience (which included ladies) heartily supporting him. Refreshments were served, but the beverages were non-intoxicants, and the members were not invited to study the subject by means of personal experiments.

The report for 1890 of the Lincoln County Hospital, shows the financial position of the hospital to be much improved. Although the year was commenced with an adverse balance of £66 4s. 1d., it has closed with a balance in favour of the institution of £285 8s. 1d. Such a happy condition of things is almost unprecedented; and it is the more satisfactory from the fact that it is entirely due to the healthy working of the hospital management; the expenditure, which had decreased in 1889 by £43 9s. 11d., having again fallen by the considerable sum of £242 8s. 10d. The subscriptions, which for the last ten years have been steadily declining in numbers and amount, have, during the past year, shown a tendency to recovery; and although the increase is not large, the Board hope that it may be regarded as evidence that the low-water mark has been reached, and that the increase will continue until the subscription list attains the level of former years.

The annual meeting of the Governors of the Leicester Infirmary was held on the 1st inst., Captain Whitmore in the chair. The report, which had previously been circulated, showed that no fewer than 3,000 in-patients and 13,614 out-patients were treated in the institution during 1890. An unsatisfactory feature in the report is that it shows a substantial increase in the average cost of each patient per week, viz., 19s. 9½d., as compared with 17s. 6½d. in the previous year. The amount received by the Treasurer from all sources during the year was £10,099 7s. 10d., while the total ordinary expenditure amounted to £10,720 2s. 6d., an increase of £1,444 4s. 3d. on that of 1889. The Chairman moved the adoption of the report, and Mr. Johnson, in seconding the motion, stated that the increased cost of working the infirmary was chiefly due to the rise in the price of coal and other provisions, and an increase in the wages account which had been necessitated by the large number of cases. The report was adopted.

Mr. John Maple has announced his willingness to give £100 to the Treasurer of the North-West London Hospital, provided that twenty others will give £50 each. As this hospital is greatly in need of additional funds, we hope Mr. Maple's condition will soon be fulfilled.

Sir E. A. Galsworthy presided, on Saturday last, at the fortnightly meeting of the Metropolitan Asylums Board, at Spring Gardens. It was reported that the number of fever patients during the past fortnight was 1,205, as against 1,295 in the preceding fortnight, showing a decrease of 90. After a long discussion, it was decided, subject to the approval of the Local Government Board, to purchase land at South Tottenham, at a cost of £12,000, for the purpose of erecting a new fever hospital.

The Royal Sea-Bathing Infirmary, at Margate, will celebrate the centenary of its existence this year. A festival dinner will be held on May 11th, at the Freemasons' Tavern, at which the Marquis of Lorne has consented to preside, in connection with which it is hoped that a substantial centenary endowment fund will be raised. This is the only hospital in England exclusively for the treatment of scrofula, a form of tuberculosis too generally prevalent in this country, and requiring for its successful treatment a bracing air and sea bathing. There is at present an annual deficit of nearly £2,000, which forms a serious obstacle to the work of the institution, and which has necessitated the closing of two wards.

The annual meeting of the Governors of the Norfolk and Norwich Hospital was held on the 9th inst., when the Lord Bishop presided. There was a very large attendance due chiefly to the interest excited by the announcement that the Lady Superintendent had joined the Roman Catholic Church. A resolution had been carried by the Board by a majority of 14 to 2, that the interests of the hospital would be consulted by Miss Adam's resignation. No one rising to speak to the resolution, the Bishop suggested the formation of a committee to consider the case in all its bearings and to make a report to a specially convened meeting of Governors. The speakers, chiefly taking their cue from the Chairman, expressed their views with judgment and moderation. There seemed to be present no disposition to aggravate sectarian feeling, but, on the contrary, to allay it. No one seemed anxious to move a resolution one way or another, and after a short and amicable debate the matter dropped. The annual report of the institution is in every way satisfactory, and shows growth both in the usefulness and popularity of the hospital.

